# The effect of low central venous pressure on hepatic surgical field bleeding and serum lactate in patients undergoing partial hepatectomy: a prospective randomized controlled trial

**DOI:** 10.1186/s12893-020-0689-z

**Published:** 2020-02-04

**Authors:** Ling Yu, Hongwei Sun, Huangmo Jin, Hongyu Tan

**Affiliations:** Department of Anesthesiology, Key laboratory of Carcinogenesis and Translational Research (Ministry of Education/Beijing), Peking University Cancer Hospital & Institute, Beijing, 100142 China

**Keywords:** Blood loss, Hepatic surgical field bleeding, Partial hepatectomy, Portal triad clamping, Serum lactate, Fluid restriction

## Abstract

**Background:**

This prospective randomized controlled study was designed to evaluate the effect of fluid restriction alone versus fluid restriction + low central venous pressure (CVP) on hepatic surgical field bleeding, intraoperative blood loss, and the serum lactate concentration in patients undergoing partial hepatectomy.

**Methods:**

One hundred forty patients undergoing partial hepatectomy with intraoperative portal triad clamping were randomized into a fluid restriction group (Group F) or fluid restriction + low CVP group (Group L). Both groups received limited fluid infusion before the liver lesions were removed. Ephedrine was administered if the systolic blood pressure (SBP) decreased to <90 mmHg for 1 min. When the urine output was <20 ml/h or the SBP was <90 mmHg for 1 min more than three times, an additional 200 ml of crystalline solution was quickly infused within 10 min. In addition to fluid restriction, patients in Group L received continuous nitroglycerin and esmolol infusion to maintain a low CVP. The duration of portal triad clamping, frequency of additional fluid infusion, frequency of ephedrine administration, intraoperative blood loss, extent of liver resection, and bleeding score of the hepatic surgical field were recorded. Arterial blood gas analysis was performed before anesthesia (T1), after liver dissection and immediately before liver resection (T2), 10 min after removal of the liver lesion (T3), and before the patient was discharged from the postanesthesia care unit (T4).

**Results:**

Being in the fluid restriction Group (Group F) (odds ratio = 5.04) and cirrhosis (odds ratio = 3.28) were risk factors for hepatic surgical field bleeding. Factors contributing to intraoperative blood loss were the operation time, duration of portal triad clamping, and extent of resection. No significant between-group difference was observed for blood loss or blood transfusion. The serum lactate concentration peaked at T3 in both groups.

**Conclusions:**

Maintaining a lower CVP during hepatectomy provides an optimal surgical field but has no significant effect on intraoperative blood loss. Moreover, lower CVP does not increase the serum lactate concentration.

**Trial registration:**

“A comparative study of the effect fluid restriction and low CVP pressure on the oozing of blood in liver wounds and blood lactate in patients undergoing partial hepatectomy” was prospectively registered as a trial (registration number: ChiCTR-INR-17014172, date of registration: 27 December 2017).

## Background

Eighty-five percent of medical centers choose to restrict fluid input to maintain low central venous pressure (CVP) and thus reduce intraoperative blood loss during hepatectomy [1]. Low pressure in the inferior vena cava also decreases pressure in the hepatic veins and hepatic sinus, which helps reduce blood loss during partial hepatectomy. Lowering the pressure in the central vein to <5 mmHg can reduce the amount of bleeding [2–5]. A recently published retrospective study showed that the absence of CVP monitoring during liver surgery had no effect on intraoperative blood loss [6]. In another study, a group of anesthesiologists evaluated 984 patients and found that fluid restriction alone was sufficient to reduce intraoperative blood loss while removing the left or right lobe for living donation, suggesting that a lower CVP is not essential for lower blood loss [7]. Moreover, absolute fluid restriction before the removal of liver lesions does not further reduce blood loss compared with relative fluid restriction [8]. Whether lower CVP can decrease blood loss in patients undergoing partial hepatectomy remains unclear [9]; therefore, further study of this controversial issue is necessary.

Hepatic blood flows into the liver through the portal vein and hepatic artery. To reduce intraoperative bleeding, many surgeons choose to obstruct blood flow to the liver using portal triad clamping (PTC), in which the hepatic artery, portal vein, and bile duct are temporarily clamped during partial hepatectomy [1, 9–12]. A previous study showed that lower CVP induced by milrinone improved the surgical field in patients undergoing living donor hepatectomy without PTC [13]. However, the effect of low CVP on surgical field bleeding of the incised liver surface during PTC remains unclear.

The arterial serum lactate concentration reflects the body’s oxygen saturation and perfusion, and a higher early lactate clearance rate shortens patients’ hospital stay [14, 15]. Warm ischemia and subsequent ischemia–reperfusion in the liver cells induced by PTC can increase the serum lactate concentration [10–12]. Studies have shown that when the serum lactate concentration is > 6 mmol/L after liver resection, the 90-day postoperative mortality rate increases significantly to 28%; however, the mortality rate is only 0.7% when the serum lactate concentration is < 2 mmol/L [16].

The current study was a prospective randomized controlled trial involving patients who underwent partial hepatectomy. Fluid restriction was performed for all patients before the liver lesions were removed. CVP was reduced by using nitroglycerin and esmolol together with fluid restriction. We investigated the effect of fluid restriction + lower CVP versus fluid restriction alone on bleeding from the incised liver surface, intraoperative blood loss, and the serum lactate concentration.

## Methods

The ethics committee of Peking University Cancer Hospital & Institute, Beijing, China approved this study in December 2017 (protocol number: 2017KT107). This trial was registered at the Chinese Clinical Trial Registry (registration number: ChiCTR-INR-17014172).

### Population and randomization

**Fig. 1:**
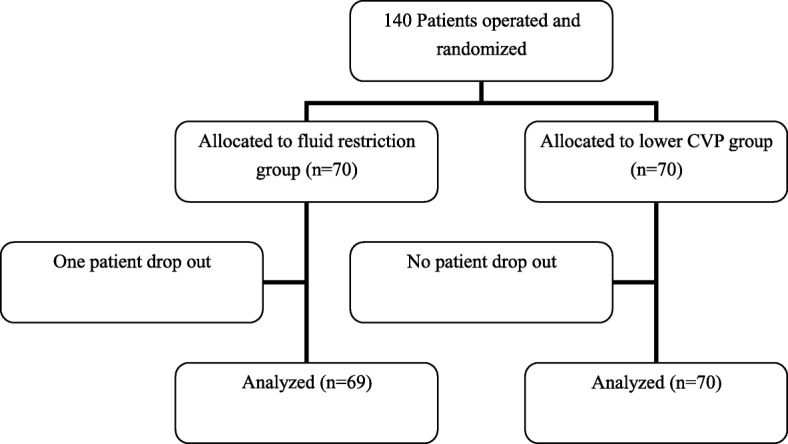
Consort diagram

In total, 140 patients who underwent elective partial hepatectomy in our hospital by an open procedure with intraoperative PTC were enrolled in this study from December 2017 to April 2019. Written informed consent was obtained from each participant before surgery. Patients aged 17 to 70 years without cirrhosis or with cirrhosis graded below Child–Pugh class A were eligible to participate in this study. Patients with a history of hypertension, a history of diabetes with uncontrolled hypertension or blood glucose, or a preoperative hemoglobin concentration of <9 g/dl were excluded from this study. Patients who underwent repeat hepatectomy or concurrent surgery were also excluded from this study. The patients were randomly divided into two groups in a 1:1 ratio: the fluid restriction group (Group F) and the fluid restriction + low CVP group (Group L). The randomization was performed by a data manager who was not involved in the patient eligibility assessment and recruitment. The randomization sequence was created using a permuted block design with SAS version 9.4 (SAS Institute, Cary, NC, USA). The anesthesiologist enrolled the participants and assigned them to the interventions, and allocation was concealed from the surgeons. One patient was withdrawn from the study because of laceration and hemorrhage of the inferior vena cava (Fig. 1).

### Intervention

Both groups were treated with the same fluid regimen and anesthetic protocol. Fluid infusion was restricted before removal of the liver lesions, and the infusion rate was accelerated after liver resection in all patients. Patients in Group L underwent intravenous administration of nitroglycerin and esmolol using an infusion pump to maintain a low CVP during removal of the liver lesions. The nitroglycerin and esmolol were reduced or discontinued when the CVP reached 0 mmHg or when the systolic blood pressure (SBP) was 90 to 100 mmHg. No nitroglycerin or esmolol was infused after removal of the liver lesions in both groups. Patients in Group F did not receive nitroglycerin or esmolol to control CVP.

### Fluid regimen and anesthetic protocols

All patients underwent jugular vein or subclavian vein catheterization the day before surgery, and the position of the catheter was determined using a chest radiograph. Once the patient was in the operating room, monitoring was performed using electrocardiography, pulse oximetry, end-tidal carbon dioxide, invasive arterial pressure, CVP, urine output, and the bispectral index. Fluid infusion was restricted at 6 ml·kg^− 1^·h^− 1^ from the moment the patient arrived in the operating room to immediately before the liver lesions were removed. Ephedrine (6 mg) was administered if the SBP decreased to <90 mmHg for 1 min. When the urine output rate was <20 ml/h or the SBP was <90 mmHg for 1 min more than three times, an additional infusion of 200 ml of crystalline solution was quickly infused within 10 min. Packed red blood cells were transfused if the hemoglobin concentration decreased to <8 g/dl. When the liver lesions were removed, the fluid infusion rate was no longer limited, and the infusion was accelerated by 1000 to 1500 ml/h. The starting infusion rates of nitroglycerin and esmolol during partial hepatectomy in Group L were 0.5 μg·kg^− 1^·min^− 1^ and 25 μg·kg^− 1^·min^− 1^, respectively. If the speed of administration of the vasoactive agents required adjustment, nitroglycerin was added or decreased by 0.1 μg·kg^− 1^·min^− 1^, and esmolol was increased or decreased by 5 μg·kg^− 1^·min^− 1^. Anesthesia was induced with 1 mg of intravenous midazolam, 0.4 to 0.5 μg/kg of intravenous sufentanil, 0.2 mg/kg of intravenous cisatracurium, and 2.0 to 2.5 mg/kg of intravenous propofol. General anesthesia was maintained with 1% sevoflurane with intravenous propofol and remifentanil infusions to maintain the bispectral index at 40 to 50. The oxygen inhalation flow rate was 50% during anesthesia, and the ventilator was adjusted to maintain the end-tidal carbon dioxide at 35 to 45 mmHg. At the end of the operation, the patient was transferred to the postanesthesia care unit and then transferred to the ward after extubation.

### Data collection and surgical procedures

The patients’ demographic data, operation time, frequency of ephedrine administration, frequency of additional 200 ml of crystalline solution infusion, intraoperative urine output and blood loss, and overall duration of PTC were recorded. Adverse events such as postoperative delirium, delayed recovery, urine output of <20 ml/h, and emergence agitation were recorded. The bleeding score of the hepatic surgical field (Table 1) was determined as described by Fromme et al. [17] and Das et al. [18]. The same surgeon, who was blinded to the group assignment, assessed hepatic surgical field bleeding.

**Table 1:** Bleeding score of the hepatic surgical field

Bleeding score	Group F (*n* = 69)	Group L (*n* = 70)	*X*^2^	*P*
0 = no bleeding (n)	2	8	–	–
1 = minor bleeding, no aspiration required (n)	7	22	–	–
2 = minor bleeding, aspiration required (n)	34	28	–	–
3 = minor bleeding, frequent aspiration required (n)	12	6	–	–
4 = moderate bleeding, visible only aspiration (n)	14	6	–	–
5 = severe bleeding, frequent aspiration required, very hard to perform surgery (n)	0	0	17.133	0.002

Group F: the fluid restriction group; Group L: the fluid restriction + low CVP group

In the present study, the use of PTC was determined according to the location and size of the liver mass during surgery. Therefore, the choices of the surgical procedure and PTC were not related to the purpose of the study. Intermittent PTC was used in cycles of 15/5 min for clamping/unclamping of the portal triad. Arterial blood gas analysis was performed before anesthesia (T1), after liver dissection and immediately before liver resection (T2), 10 min after removal of the liver lesions (T3), and before the patient was discharged from the postanesthesia care unit (T4). The maximum and minimum CVP were recorded during PTC, and the mean was calculated. Blood pressure was recorded every 5 min during PTC, and the mean SBP and mean arterial pressure (MAP) were calculated.

The same surgical team performed the liver resections in both groups. Intermittent PTC using a vascular tourniquet was applied during parenchymal resection whenever needed. Liver resection was performed by ultrasonic dissection for parenchymal transection, ligation or clamping of blood vessels, and coagulation dissectors for vessel sealing. The same hepatic parenchymal transection technique was used in all patients. The extent of liver resection was divided into major hepatectomy and minor hepatectomy, with major hepatic resection defined as resection of three or more segments. The extent of liver resection was assessed by hepatobiliary surgeons.

### Outcome and sample size calculation

The primary outcome of interest was the bleeding score of the hepatic surgical field. The secondary outcomes were the serum lactate concentration, intraoperative blood loss, frequency of additional infusions of 200 ml of crystalline solution, intraoperative urine output, duration of PTC, mean CVP, and mean SBP during PTC.

The sample size was estimated using PASS software, version 11.0 (NCSS, LLC, Kaysville, UT, USA). The sample size was calculated using a test for two ordered categorical variables with a randomization ratio of 1:1, two-sided significance level of 0.05, and power of 0.8. According to the data in the trials by Ryu et al. [13] and Das et al. [18], the control group category proportions were 0.1, 0.2, 0.4, 0.2, and 0.1, and the logarithm of the odds radio of the treatment group was assigned a value of 0.9. We determined that 64 patients would be required in each group, and we recruited 70 patients in each group to address patients possibly leaving the study.

### Statistical analysis

SPSS version 18.0 for Windows (SPSS Inc., Chicago, IL, USA) was used for data processing. Normally distributed measurement data are expressed as mean ± standard deviation, and non-normally distributed measurement data are expressed as median and interquartile range. Multivariate ordered logistic regression analysis was employed to study the relationships between potential risk factors and the bleeding score of the hepatic surgical field. Multivariate linear regression analysis was used to identify the relationship between intraoperative blood loss and influencing factors. Selection of variables was based on the literature and physiologically and clinically valid models of the phenomenon being studied. Repeated-measures analysis of variance was used for within-group comparisons of differences in the serum lactate concentration. The independent-sample *t*-test was performed for between-group analysis of the serum lactate concentration, hemoglobin concentration, mean SBP during PTC, mean MAP during PTC, and operation time. The Mann–Whitney *U* test for two samples was performed to compare the extent of liver resection, type of liver resection, mean CVP during PTC, duration of PTC, frequency of ephedrine administration, frequency of additional fluid infusion, urine output, intraoperative blood loss, and blood transfusion between the groups. Categorical data were compared using the *X*^2^ test. A *P* value of < 0.05 was considered statistically significant.

## Results

One patient in Group F dropped out of the study; thus, data from 69 patients in Group F and 70 patients in Group L were available for analysis. The disease types were primary hepatocellular carcinoma (67 patients) and liver metastasis from colorectal cancer (73 patients). The study group comprised 33 women and 107 men aged 55.1 ± 10.5 years (range, 26–70 years). There were no statistically significant differences in the demographic data between the two groups (Table 2). The types of liver resection are shown in Table 3. The type of liver resection for multiple nodules was determined by the location of the liver segment in which the largest mass was located. There was no significant difference in the extent of liver resection (*Z* = − 0.069, *P* = 0.945) or the type of liver resection (*Z* = − 0.944, *P* = 0.345) between the two groups.

**Table 2:** Patients’ demographic data in the two groups

	Group F (*n* = 69)	Group L (*n* = 70)
Age (year)	55.3 ± 9.6	54.9 ± 11.2
Male/female (n)	56/13	50/20
Primary liver carcinoma/Liver metastases (n)	32/37	34/36
Chronic HBV infection	19	17
Chronic HCV infection	1	1
Alcohol abuse	2	1
Presence of cirrhosis (n)	18	19
Comorbidities: Hypertension/Coronary artery disease/Diabetes/ chronic nephritis/Asthma/ Alcohol abuse (n)	13/5/8/0/1/1	17/3/4/1/0/2

*HBV* Hepatitis B virus, *HCV* Hepatitis C virus; Group F: the fluid restriction group; Group L: the fluid restriction + low CVP group

**Table 3:** Type of liver resection

	Group F	Group L
Major liver resection	28	28
Left hepatectomy (n)	8	6
Extended Left hepatectomy (n)	3	0
Right hepatectomy (n)	8	12
Extended right hepatectomy (n)	1	3
Central hepatectomy (n)	8	7
Minor liver resection	41	42
Left-sided hepatectomy (II, III segments) (n)	5	3
Right-sided hepatectomy (VI, VII segments (n)	20	20
Central hepatectomy (IV, V, VII segments) (n)	16	19

Group F: the fluid restriction group; Group L: the fluid restriction + low CVP group

Intraoperative and anesthetic data are shown in Table 4. All patients in both groups accepted the PTC maneuver. Ephedrine was administered significantly more frequently and the mean CVP and SBP during PTC were significantly lower in Group L than in Group F. No significant between-group differences were observed for the frequency of additional fluid infusion, duration of PTC, operation time, intraoperative blood loss, blood transfusion, or intraoperative urine output. The bleeding score of the hepatic surgical field was significantly different between the two groups (*X*^2^ = 17.133, *P* = 0.002). Postoperative delirium, delayed recovery, oliguria, and emergence agitation did not occur in any patient in either group.

**Table 4:** Intraoperative and anesthetic data in the two groups

	Group F (*n* = 69)	Group L (*n* = 70)	*t* or Z value	*P* value
Duration of all PTC (mins)	25.0 (15.5, 37.5)	25.5 (15, 34.5)	−0.089	0.929
Blood transfusion/no blood transfusion (n)	4/69	2/70	−0.937	0.349
Operation time (mins)	189.6 ± 59.6	187.8 ± 54.5	0.946	0.861
Urine output (ml)	200 (100, 300)	200 (100, 250)	−0.637	0.524
Blood loss (ml)	200 (125, 400)	200 (150, 425)	−0.133	0.894
CVP during PTC (mm Hg)	4.0 (3, 5)	2.5 (1.5, 3.5)	−5.064	< 0.001*
SBP during PTC (mm Hg)	109.6 ± 11.0	103.1 ± 10.8	3.518	0.001*
MAP during PTC (mm Hg)	78.3 ± 8.7	74.6 ± 7.6	2.630	0.010*
Frequency of additional fluid infusion (0/1/more than 2) (n)	42/19/8	38/24/8	−0.697	0.486
Frequency of ephedrine (0/1/2/more than 3) (n)	41/12/14/2	13/20/15/22	−5.199	< 0.001*

The data are presented as median and interquartile range (M (P25, P75) or mean ± standard deviation or numbers of patients. Group F: the fluid restriction group; Group L: the fluid restriction + low CVP group; *CVP* Central venous pressure, *SBP* Systolic blood pressure, *PTC* Portal trail clamping, *MAP* Mean arterial pressure.* *P* < 0.05

The factors contributing to intraoperative blood loss by level of importance were the operation time, duration of PTC, and extent of resection. No significant correlation was detected between intraoperative blood loss and other factors such as the presence of cirrhosis, mean CVP during PTC, bleeding score of the hepatic surgical field, SBP during PTC, or frequency of ephedrine administration. The coefficients of linear regression are shown in Table 5.

**Table 5:** Coefficients^a^ of multivariate linear regression

	Unstandardized Coefficients		Standardized Coefficients	*T* value	*P* value
	B	Std. Error	Beta		
Constant	− 280.338	206.928		−1.335	.178
No cirrhosis = 1; presence of cirrhosis = 2	−17.499	37.404	−.032	−.468	.641
Mean CVP value during PTC	−2.983	9.794	−.021	−.305	.761
Frequency of ephedrine	15.406	13.675	.086	1.127	.262
Operation time	1.766	.313	.423	5.637	.000*
Duration of all PTC	3.618	1.127	.229	3.209	.002*
Group F = 1; group L = 2	−1.347	39.607	−.003	−.034	.973
Minor hepatectomy = 1; major hepatectomy = 2	101.628	32.958	.204	3.084	.003*
SBP during PTC	.586	1.536	.027	.381	.704
Bleeding score of surgical field	22.289	16.281	.100	1.369	.173

^a^: dependent variable: intraoperative blood loss. Group F: the fluid restriction group; Group L: the fluid restriction + low CVP group; CVP: central venous pressure; SBP: systolic blood pressure; PTC: portal trail clamping. * *P* < 0.05

**Fig. 2:**
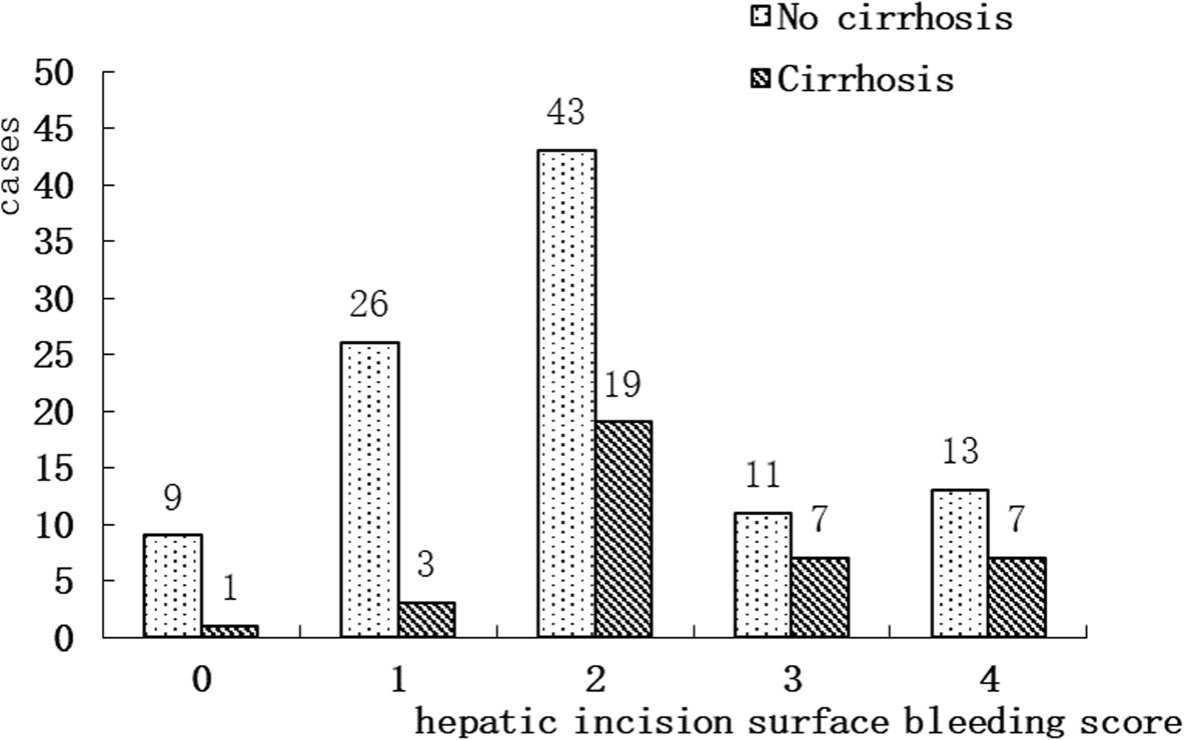
Comparison of bleeding score of the hepatic surgical field in patients with or without cirrhosis for all patients (*n* = 139)

The bleeding score of the hepatic surgical field during PTC in both groups is shown in Table 1. Ordered logistic regression analysis was performed with the bleeding score of the hepatic surgical field as the outcome variable, and the results are shown in Table 6. The odds ratio describes the odds of a one-category increase in the outcome for a 1-unit change in the explanatory variables. The risk of hepatic surgical field bleeding was higher in Group F than Group L (odds ratio = 5.04). Moreover, the risk of hepatic surgical field bleeding increased in the presence of cirrhosis (odds ratio = 3.28). A comparison of hepatic surgical field bleeding in patients with and without cirrhosis is shown in Fig. 2.

**Table 6:** Results of the ordered logistic regression analysis of hepatic surgical field bleeding

Risk factor	Odds ratio	95% CI	*P* value
Primary HCC = 1; liver metastasis = 2	1.68	0.86----3.49	0.159
No cirrhosis = 1; presence of cirrhosis = 2	3.28	1.39----7.76	0.007*
Minor hepatectomy =1; major hepatectomy =2	1.48	0.76----2.73	0.249
Frequency of ephedrine dosing	0.99	0.73----1.33	0.928
Frequency of additional fluid	1.32	0.84----2.08	0.236
SBP during PTC	0.97	0.94----1.01	0.107
Duration of PTC	1.02	1.00----1.04	0.100
Group F	5.04	2.38----10.69	0.000*
Group L	1		

Group F: the fluid restriction group; Group L: the fluid restriction + low CVP group; *HCC* Hepatocellular carcinoma, *SBP* Systolic blood pressure, *PTC* Portal trail clamping. * *P* < 0.05

**Fig. 3:**
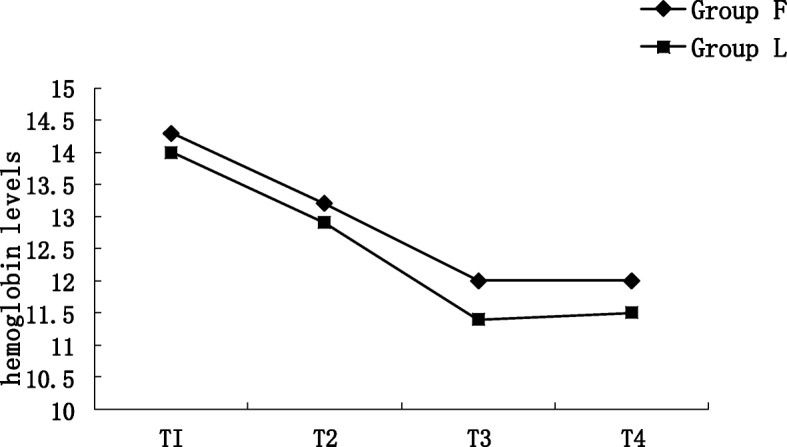
The hemoglobin levels in 2 groups. T1: before anesthesia; T2: after liver dissection and immediately before liver resection; 10 min after removal of the liver lesion (T3), and before the patient was discharged from the postanesthesia care unit (T4)

The hemoglobin concentrations at T1, T2, T3, and T4 are shown in Fig. 3. No significant difference in the hemoglobin concentration was observed at any time point between the two groups.

**Fig. 4:**
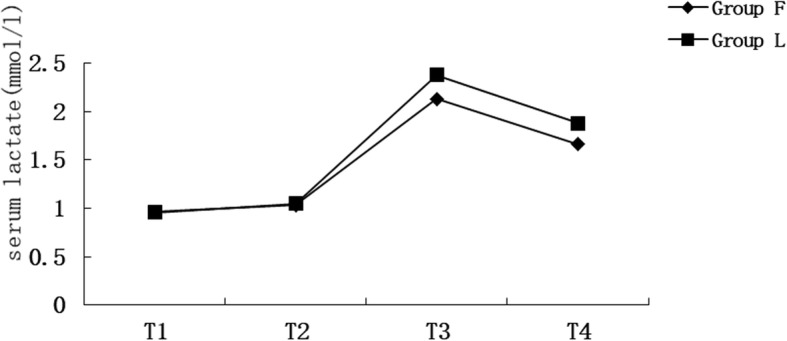
The serum lactate level in 2 groups. T1: before anesthesia; T2: after liver dissection and upon starting liver resection; T3: 10 min after removal of the liver lesion (T3), and before the patient was discharged from the postanesthesia care unit (T4)

The results of the repeated-measures analysis of variance showed that the serum lactate concentration was significantly different at T1, T2, T3, and T4 in Group F [*F* (1.542, 104.829) = 65.121, *P* < 0.001] and Group L [*F* (1.773, 122.356) = 135.232, *P* < 0.001]. The serum lactate concentration peaked at T3 in both groups. The independent-sample *t*-test showed no significant between-group difference in the serum lactate concentration at any time point (Fig. 4).

## Discussion

The relationship between low CVP and blood loss remains controversial [2–5, 19, 20]. The methods used to maintain a lower CVP are placing the patient in the Trendelenburg posture, administering diuretics or vasodilators, limiting the infusion speed, and clamping the infrahepatic vena cava [4]. Notably, changes in body position markedly decrease CVP but not portal or hepatic venous pressure. Manipulating a patient into the Trendelenburg position to reduce blood loss during liver surgery may not be effective [21]. Our results are consistent with the findings from other studies [6, 7, 22, 23] showing that fluid restriction alone reduced blood loss during liver surgery. No significant difference was observed for intraoperative blood loss, transfusion requirement, or hemoglobin concentration between the two groups in this study.

Less bleeding of the liver surface occurs in living liver donors with a low CVP undergoing hepatectomy, leading to a drier and cleaner surgical field [13]. Blood loss during hepatectomy is caused by the destruction of vessels and venous bleeding from the sectioned liver surface after blood flow into the liver (including through the hepatic artery and portal vein) has been obstructed. To decrease the influence of surgical techniques on intraoperative blood loss and the bleeding score of the surgical field, all procedures in this study were performed by the same surgical team. With technological advances in surgery that allow for better control of blood vessels during hepatic resection, bleeding in the hepatic surgical field depends mainly on the pressure in the hepatic sinus. When the pressure in the inferior vena cava is lowered, pressure in the hepatic vein and sinus also decrease. This may explain why a low CVP can reduce the severity of bleeding from the incised liver surface and improve surgical field visibility.

In the present study, intraoperative blood loss was associated with the extent of liver resection, duration of PTC, and operation time, but blood loss was not related to the severity of bleeding in the hepatic surgical field. The median duration of PTC was 25 min, which was only approximately 13% of the mean operation time (188 min) in this study. Blood loss caused by oozing from the resected liver surface during PTC had little effect on intraoperative blood loss.

When decreasing CVP using nitroglycerin and esmolol, SBP decreased by approximately 10 mmHg in Group L compared with Group F. However, neither the severity of bleeding in the hepatic surgical field nor the intraoperative blood loss was related to arterial blood pressure in this study. Deliberately inducing hypotension by lowering arterial blood pressure can decrease the degree of bleeding in areas of the surgical field other than the liver surface [17, 18, 24]. In the present study, lower arterial blood pressure was not associated with the bleeding score of the surgical field or intraoperative blood loss, possibly because 60 to 80% of the blood supply is from the portal vein. After clamping the hepatic artery, which supplies 20 to 40% of the blood, the effect of arterial pressure on mitigating hepatic surgical field bleeding decreased.

The risk of an increase in the hepatic surgical field bleeding score by one or more grades increased 3.28-fold in the presence of cirrhosis in this study. The risk factors for massive bleeding (>3000 ml) during major hepatectomy are not associated with cirrhosis [25]. However, the presence of cirrhosis is a risk factor for blood loss and necessitates blood transfusion in patients undergoing hepatectomy [26].

The main limitation of this study is that we could not accurately calculate blood loss to the milliliter level during removal of the liver lesions. We found no method to accurately measure the amount of blood loss during PTC clinically. However, we used the bleeding score of the surgical field, which relied on the doctor’s assessment. Surgical field bleeding from the incised liver surface was evaluated by the same surgeon, who was blinded to the group assignments. Therefore, we believe that difficulties encountered in accurately assessing bleeding do not affect the interpretation of the results.

Intraoperative fluid restriction has no effect on the blood lactate concentration [27]; similarly, absolute fluid restriction with a low CVP during liver resection has no effect on the serum lactate concentration [8]. Control of hypotension during surgery, including by the combined use of nitroglycerin and esmolol, does not increase the blood lactate concentration, indicating no adverse effects on organ or tissue perfusion [24, 28]. In the present study, ephedrine was more frequently administered intraoperatively in Group L; however, no significant difference was observed in the serum lactate concentration between the groups. Therefore, our results suggest that a lower CVP does not result in an increased serum lactate concentration. Our results may be related to tight monitoring of the urine output to > 20 ml/h and the SBP to > 90 mmHg in all patients.

Our results suggest that the serum lactate concentration in both groups peaked at 10 min after resecting the liver lesions. Pietsch et al. [10] showed that the highest serum lactate concentration in patients undergoing PTC occurred at 10 min after opening the liver hilus, which is consistent with our study. In our study, liver blood flow was restored and the fluid infusion rate was increased after removing the liver lesions. This was associated with decreased early blood lactate concentrations in both groups, suggesting improved tissue perfusion and oxygenation [14].

## Conclusions

Maintaining a lower CVP by fluid restriction and administering nitroglycerin and esmolol during PTC provides an optimum surgical field but has no significant effect on intraoperative blood loss compared with limiting fluid infusion alone. Moreover, a lower CVP does not increase the serum lactate concentration when urine output and systolic blood pressure are maintained.

## Data Availability

The datasets generated during the current study are available in the http://www.medresman.org/login.aspx, and the number is ChiCTR-INR-17014172. The data is available from the corresponding author under reasonable request. The email of corresponding author is maggitan@yeah.net.

## Abbreviations

CVP: Central venous pressure
MAP: Mean arterial pressure
PTC: Portal triad clamping
SBP: Systolic blood pressure
